# Genome‐wide siRNA library screening identifies human host factors that influence the replication of the highly pathogenic H5N1 influenza virus

**DOI:** 10.1002/mlf2.12168

**Published:** 2025-02-24

**Authors:** Guangwen Wang, Li Jiang, Jinliang Wang, Qibing Li, Jie Zhang, Fandi Kong, Ya Yan, Yuqin Wang, Guohua Deng, Jianzhong Shi, Guobin Tian, Xianying Zeng, Liling Liu, Zhigao Bu, Hualan Chen, Chengjun Li

**Affiliations:** ^1^ State Key Laboratory for Animal Disease Control and Prevention, Harbin Veterinary Research Institute Chinese Academy of Agricultural Sciences Harbin China

**Keywords:** genome‐wide siRNA library screening, GO analysis, H5N1 influenza virus, host cellular factor, reactome pathway analysis

## Abstract

The global dissemination of H5 avian influenza viruses represents a significant threat to both human and animal health. In this study, we conducted a genome‐wide siRNA library screening against the highly pathogenic H5N1 influenza virus, leading us to the identification of 457 cellular cofactors (441 proviral factors and 16 antiviral factors) involved in the virus replication cycle. Gene Ontology term enrichment analysis revealed that the candidate gene data sets were enriched in gene categories associated with mRNA splicing via spliceosome in the biological process, integral component of membrane in the cellular component, and protein binding in the molecular function. Reactome pathway analysis showed that the immune system (up to 63 genes) was the highest enriched pathway. Subsequent comparisons with four previous siRNA library screenings revealed that the overlapping rates of the involved pathways were 8.53%–62.61%, which were significantly higher than those of the common genes (1.85%–6.24%). Together, our genome‐wide siRNA library screening unveiled a panorama of host cellular networks engaged in the regulation of highly pathogenic H5N1 influenza virus replication, which may provide potential targets and strategies for developing novel antiviral countermeasures.

## Impact statement

A genome‐wide siRNA library screening was conducted against the highly pathogenic H5N1 influenza virus, and 457 candidate genes were identified and delineated by Gene Ontology (GO) and reactome pathway analysis. Of these, 41 host factors had previously been revealed to be required for different stages of influenza A virus (IAV) replication, such as endosomal acidification and trafficking, fusion, nuclear import and export, and viral ribonucleoprotein (vRNP) complex activity, or to be associated with immunity and inflammatory response or other biological processes. Thus, our genome‐wide siRNA library screening comprehensively mined the host factors involved in the replication of the highly pathogenic H5N1 influenza virus, which may aid the development of host‐directed anti‐influenza drugs.

## INTRODUCTION

The influenza A virus (IAV) is an enveloped virus containing a segmented negative‐sense single‐stranded RNA genome. In addition to the severe threat posed by occasional human influenza pandemics, frequent seasonal influenza epidemics also place a severe burden on human health. Furthermore, widespread H5 and H7 avian influenza viruses cause severe damage to the poultry industry^1^, ^2^, ^3^, ^4^, ^5^, ^6^ and sporadically cross the species barrier to infect humans^7^, ^8^, ^9^, ^10^, ^11^, resulting in 939 (H5N1), 93 (H5N6), and 1568 (H7N9) cases of human infections as of November 1, 2024^12^. It is noteworthy that the use of an H5/H7 bivalent inactivated avian influenza vaccine since September 2017 in China has not only successfully controlled H7N9 avian influenza infections in poultry but also eliminated human infections^13^, ^14^. However, the constant reassortment of H5 viruses worldwide drives the continuous emergence of different virus subtypes, such as H5N1, H5N2, H5N6, and H5N8 viruses, causing numerous serious outbreaks worldwide^10^. Moreover, when dramatic antigenic change occurs between epidemic strains and vaccine strains, the vaccine seed virus must be periodically updated^14^, ^15^. In addition, IAV mutants resistant to antiviral drugs, such as matrix protein 2 (M2) blockers (amantadine and rimantadine), neuraminidase (NA) inhibitors (oseltamivir, zanamivir, and peramivir), and polymerase acidic protein (PA) inhibitor (baloxavir), weaken the clinical effectiveness of these antiviral drugs^16^, ^17^. Therefore, a better understanding of the landscape of host cellular factors involved in the replication of IAV, especially the highly pathogenic H5 virus, can provide fundamental insights for developing effective countermeasures for the prevention and treatment of influenza‐related diseases.

**Figure 1:**
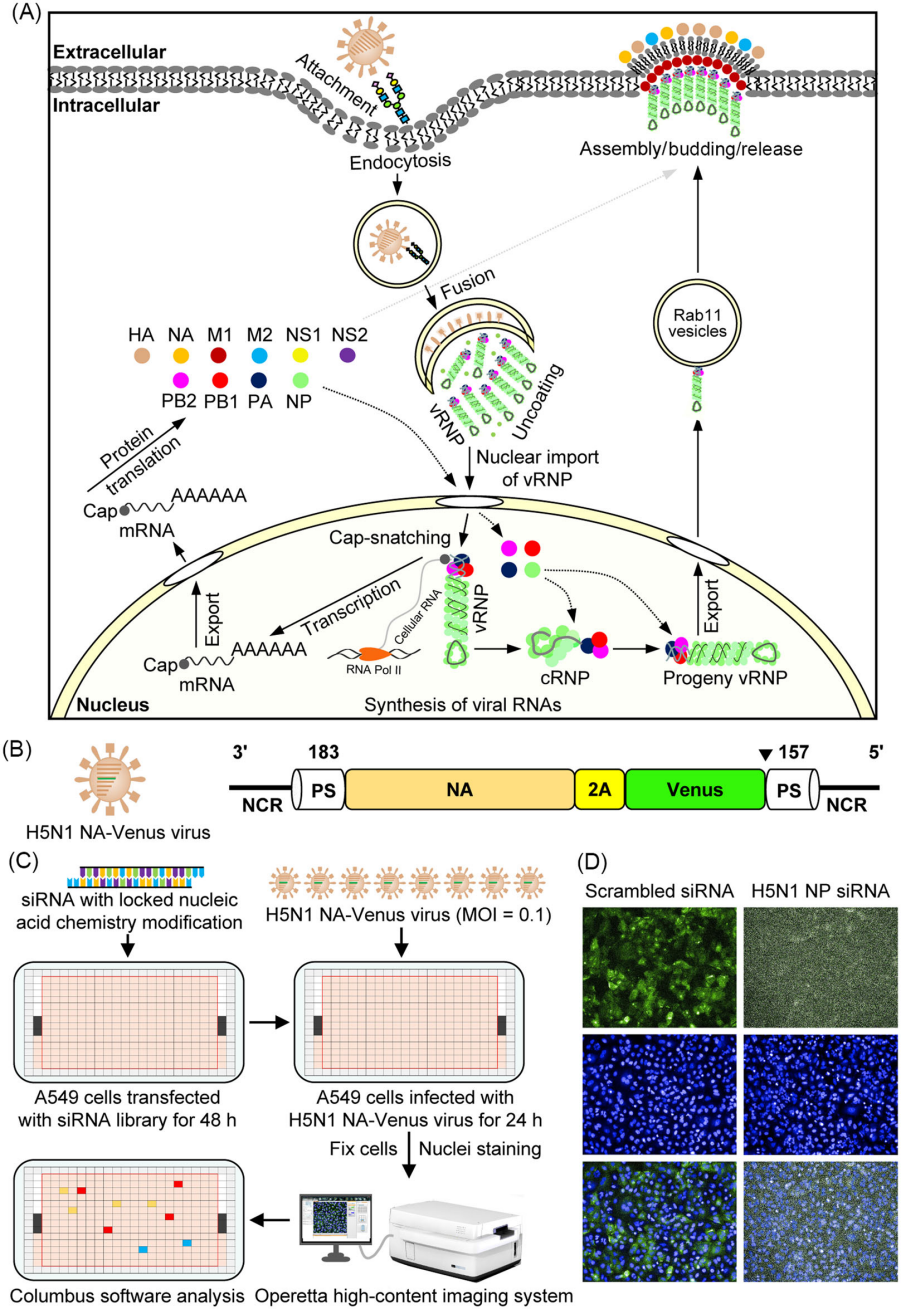
Genome‐wide siRNA library screening uncovers host factors required for the replication of the highly pathogenic H5N1 influenza virus. (A) Schematic diagram of the IAV replication cycle. (B) Schematic representation of the H5N1 NA‐Venus virus harboring a reporter neuraminidase (NA) segment integrated with the Venus gene. NCR, noncoding region; PS, packaging signal; 2A, a self‐cleaving 2A sequence of porcine teschovirus‐1; and ▼, stop codon. (C) Schematic diagram of the genome‐wide siRNA library screening for host factors that regulate H5N1 virus replication in A549 cells. The image of the Operetta high‐content screening machine was derived from the PerkinElmer manual. (D) Representative images of Venus expression in scrambled siRNA‐ or H5N1 NP siRNA‐treated A549 cells. A549 cells were transfected with siRNA targeting H5N1 NA‐Venus NP or scrambled siRNA for 48 h, and then infected with the H5N1 NA‐Venus virus (MOI = 0.1) for 24 h. Cells were then fixed with 4% PFA for 30 min, and their nuclei were stained with Hoechst 33342 for 30 min at room temperature. Images were captured using the Operetta high‐content imaging system.

IAV completes its life cycle within infected host cells through a set of critical steps that include attachment, endocytosis, fusion, uncoating, nuclear import of viral ribonucleoprotein (vRNP), synthesis of viral RNAs, viral protein translation, nuclear export of progeny vRNP, and assembly/budding/release of progeny virus (Figure 1A). For initial attachment, the viral hemagglutinin (HA) binds to sialic acid receptors present in the oligosaccharides of glycoproteins or glycolipids at the cell surface^18^. The virus then immediately initiates endocytosis through several different mechanisms: clathrin‐mediated endocytosis, clathrin‐ and caveolin‐independent endocytosis, and macropinocytosis^19^, ^20^, ^21^. The internalized viral particles are transported through early endosomes to late endosomes, where the acidic environment triggers the conformational change in HA, leading to the fusion of the endosomal membrane and the viral envelope^22^. Then, uncoating initiation accelerates M1 dispersion and the release of eight vRNPs into the cytoplasm^23^, ^24^. The vRNPs are subsequently imported into the nucleus of the infected cells through nuclear pore complexes^25^. Once in the nucleus, viral RNA transcription and replication by RNA‐dependent RNA polymerase (RdRp) produces capped and polyadenylated mRNAs, which are exported into the cytoplasm for translation into viral proteins, and also creates positive‐sense complementary RNA (cRNA) with the help of newly synthesized viral polymerases (PB2, PB1, and PA) and nucleoprotein (NP)^26^. Progeny vRNAs are synthesized from the cRNA templates, exported from the nucleus into the cytoplasm in the form of vRNPs, and finally transported to the plasma membrane via Rab11‐dependent vesicles^27^. The assembly, budding, and release of progeny virions occur at the plasma membrane, where eight vRNPs and newly synthesized viral proteins are incorporated^28^, ^29^. To date, the entire IAV life cycle has been clearly outlined, and molecular details of each step are continuously accumulating. Although some essential host factors, such as ANP32A^30^, mGluR2^31^, and SLC35A1^32^, are gradually being discovered, the landscape of host factors involved in the regulation of the IAV life cycle is still not well understood.

Genome‐wide siRNA library screening is a panoramic and highly efficient method to identify many host factors capable of regulating the IAV life cycle, which can then provide information about host cellular determinants of virus replication and uncover potential targets for developing novel antiviral countermeasures. Although four genome‐wide siRNA library screenings for IAV have been conducted, the strains used in these screenings were all low pathogenic H1N1 viruses. Given the widespread circulation of H5 influenza viruses worldwide and the severe harm that they cause to human and animal health, we conducted a genome‐wide siRNA library screening of a highly pathogenic H5N1 influenza virus strain in human lung carcinoma cells (A549) to comprehensively mine the host cellular factors and machinery involved in the virus replication cycle.

## RESULTS

### Genome‐wide siRNA library screening for host cellular factors that regulate H5N1 influenza virus replication

To conduct a complete genome‐wide siRNA library screening against the H5N1 virus, we first generated a replication‐competent Venus‐expressing H5N1 influenza virus: H5N1 NA‐Venus (Figure 1B), which stably expresses a strong Venus fluorescent signal, shows similar plaque sizes and comparable or slightly less growth titers in cell culture and embryonated chicken eggs, and has similar high pathogenicity in mice as its parental A/Anhui/2/2005 (AH05, H5N1) virus^33^. The characteristics of the H5N1 NA‐Venus virus make it an ideal reporter virus to identify host cellular factors involved in the life cycle of the H5N1 virus.

**Figure 2:**
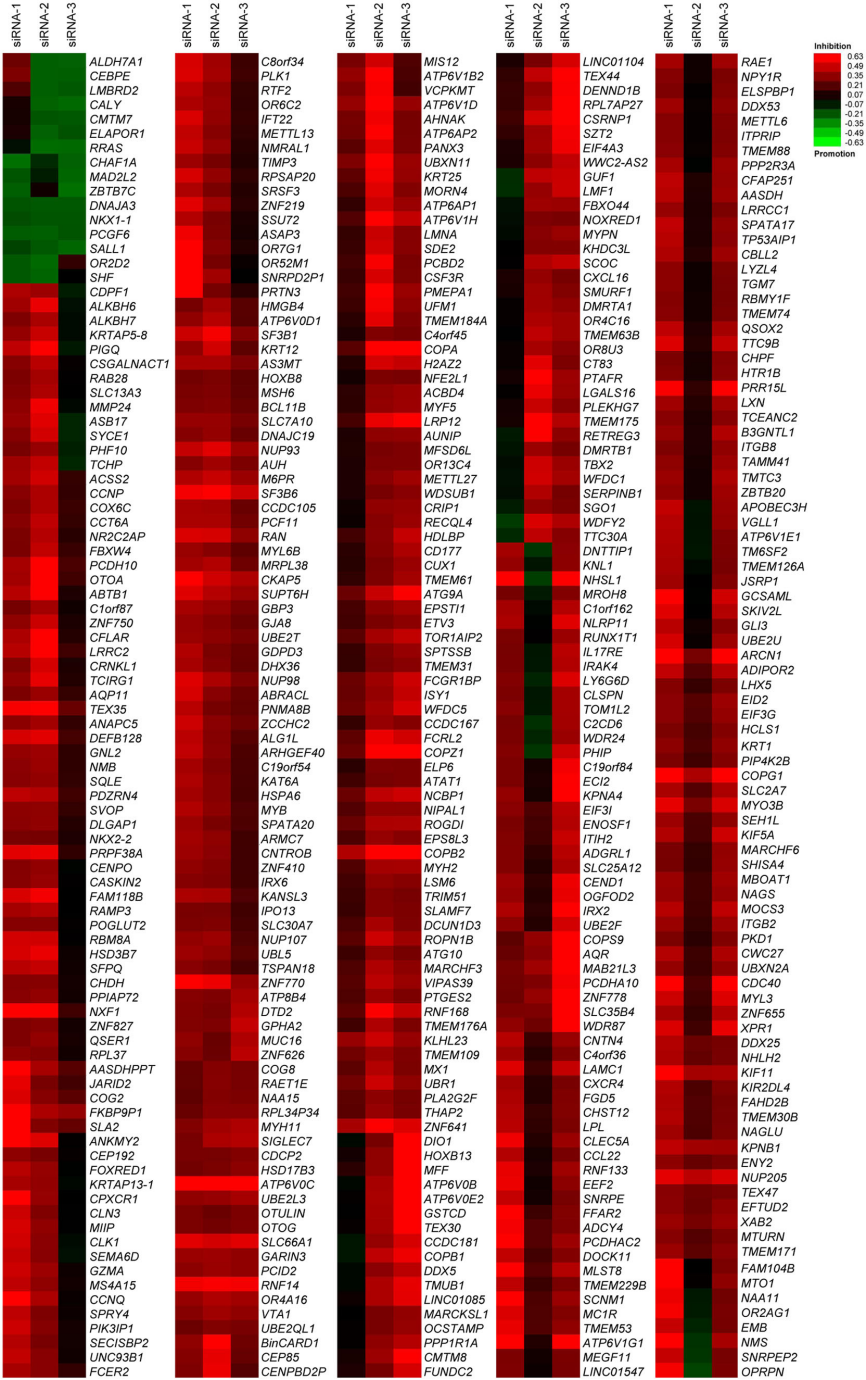
Heatmap analysis of candidate genes. The redder the color of a gene, the greater the inhibitory effect observed upon knocking down the expression of that gene using three individual siRNAs. The greener the color of a gene, the greater the promoting effect observed upon knocking down the expression of that gene using three individual siRNAs.

For high‐throughput screening (Figure 1C), A549 cells were transfected with individual siRNA within an siRNA library targeting 21,585 human genes arrayed in 284 384‐well plates for 48 h, before being infected with H5N1 NA‐Venus virus (MOI = 0.1). The infected A549 cells were fixed at 24 h postinfection and then stained with the nuclear dye Hoechst 33342. Subsequently, images were captured using an Operetta high‐content imaging system, and the infection ratio of the cells was analyzed using Columbus 2.9.1 software based on the Venus fluorescence intensity. The infection ratio in the scrambled siRNA‐transfected wells was set at approximately 70%, which guaranteed the identification of proviral host factors as well as restricting host factors. The positive control siRNA targeting H5N1 NP mRNA effectively inhibited H5N1 NA‐Venus virus replication in each screening plate, validating the reliability of the assay (Figure 1D). In the final stage of the comprehensive data analysis, we first utilized the Z‐factor, a way to examine the well‐to‐well and plate‐to‐plate reproducibility, to evaluate the quality of the screening assay. We found that the Z‐factors of all plates were ≥0.5, which indicates the excellence of the established assay (Figure S1). Subsequently, candidate genes from three independent screenings were acquired using two screening parameters: the strictly standardized mean difference (SSMD) and the inhibition ratio (IR). A cellular gene for which at least two siRNAs had an |SSMD| value ≥1.28 and an IR ≥30%, or an |SSMD| value ≥1.28 and an IR ≤−20% was designated as a proviral host factor or an antiviral host factor, respectively. Using these criteria, 441 proviral host factors and 16 antiviral host factors (total number: 457) from a total of 21,585 human genes were designated as candidate genes (Figure 2 and Table S1), which accounted for 2.12% of the genes of the entire human genome (Table 1). Interestingly, although the total gene number of the Human Genome Collection subset (11,170 genes) was greater than that of the Human Druggable Genome subset (9032 genes), its number of candidate genes (219, 1.96%) was lower than that of the Human Druggable Genome subset (225, 2.49%) (Table 1). Based on our comprehensive analysis of each library component, we found that the Human Druggable Genome subset had many genes associated with kinases, phosphatases, proteases, G protein‐coupled receptors, nuclear hormone receptors, and ion channels, which could be potential drug targets for antiviral therapies.

**Table 1:** Number of candidate genes related to three library subsets.

Library subset	Proviral factors	Antiviral factors	Total
Human Druggable Genome (9032)	219 (2.42%)	6 (0.07%)	225 (2.49%)
Human Extended Druggable Genome (1383)	11 (0.80%)	2 (0.14%)	13 (0.94%)
Human Genome Collection (11,170)	211 (1.89%)	8 (0.07%)	219 (1.96%)
Total (21,585)	441 (2.04%)	16 (0.07%)	457 (2.12%)

The percentage in the round bracket is the number of candidate genes/the number of library subsets.

### GO classifications and reactome pathway analysis

**Figure 3:**
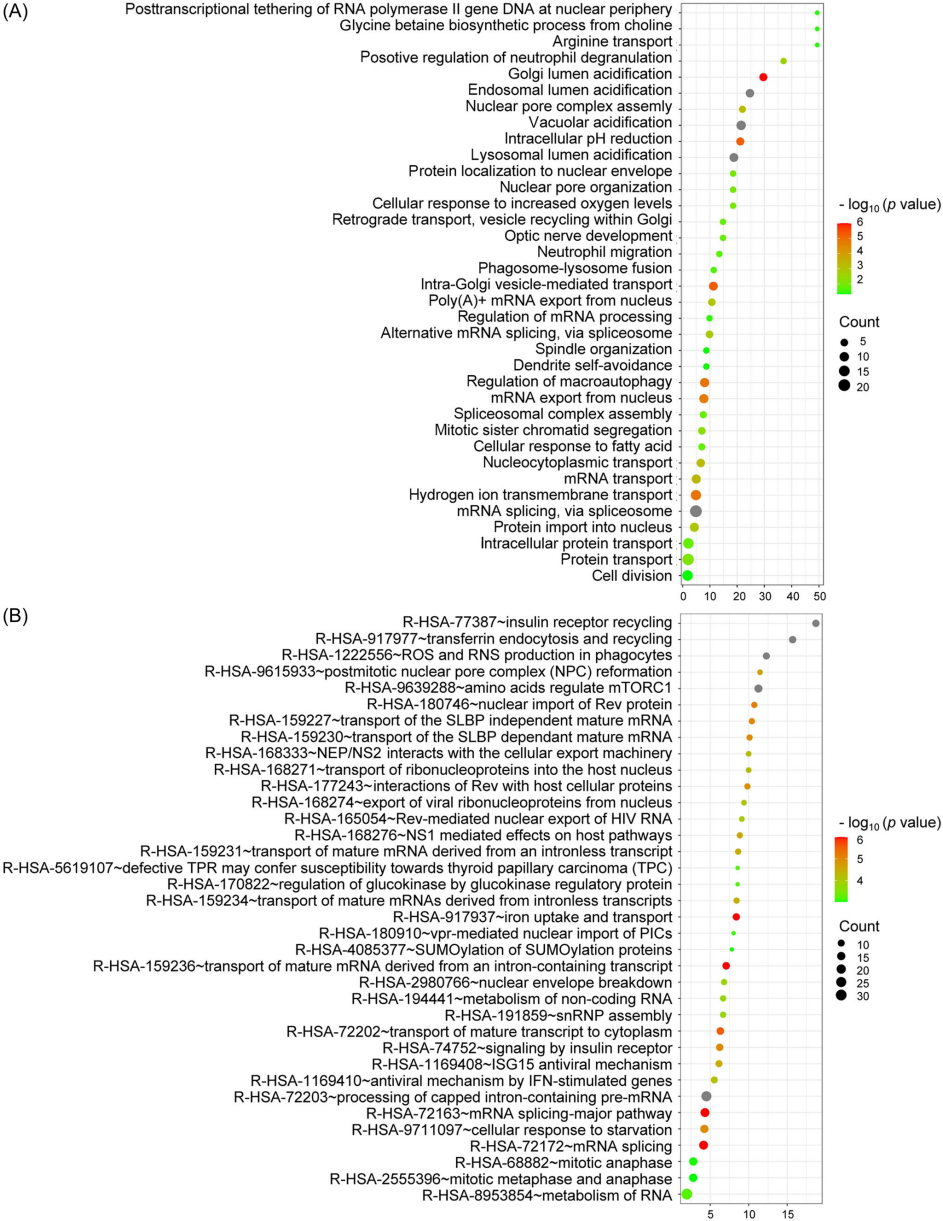
Biological process enrichment analysis and reactome pathway analysis of candidate genes. (A) Biological process classification of *p* ≤ 0.05. (B) Reactome pathway classification of *p* ≤ 0.001. The X‐axis shows the enrichment score and the Y‐axis shows the biological process classification (A) and the reactome pathway classification (B), respectively. The smaller the *p* value in the classification, the redder the bubble color; the higher the number of genes in the classification, the larger the bubble size.

To acquire an extensive and comprehensive understanding of the biological characteristics of the candidate genes, we first performed a GO functional annotation analysis using the online analytical tool DAVID (Database for Annotation, Visualization and Integrated Discovery)^34^. GO, a universally utilized gene functional enrichment database, was applied to search for enriched GO terms, such as biological process, cellular component, and molecular function^35^. In total, 389 out of the 457 candidate genes were assigned to 89 GO terms (*p* ≤ 0.05), which comprised 36 biological process terms (assigned to 102 genes), 40 cellular component terms (assigned to 288 genes), and 13 molecular function terms (assigned to 317 genes) (Figure 3A and S2, Table S2). Among them, the top four significant biological process terms were mRNA splicing via spliceosome (20 genes, *p* = 3.05E−8), protein transport (18 genes, *p* = 8.29E−3), intracellular protein transport (14 genes, *p* = 1.65E−2), and cell division (14 genes, *p* = 4.70E−2). Analysis of the GO cellular component identified the top three enrichment terms as integral component of membrane (130 genes, *p* = 1.41E−2), cytosol (130 genes, *p* = 2.12E−2), and membrane (64 genes, *p* = 4.77E−2) (Figure S2A and Table S2). The top three terms for molecular function were protein binding (301 genes, *p* = 5.10E−4), RNA binding (44 genes, *p* = 2.38E−2), and hydrolase activity (12 genes, *p* = 3.36E−2) (Figure S2B and Table S2).

To identify the regulatory pathways of the candidate genes, we performed a reactome signaling pathway enrichment analysis with DAVID. Reactome is a database of signaling and metabolic molecules in which their relationships are organized into biological pathways and processes^36^. We found that the top four pathways were those associated with the immune system (63 genes, *p* = 4.77E−3), metabolism of RNA (30 genes, *p* = 5.77E−4), processing of capped intron‐containing pre‐mRNA (25 genes, *p* = 1.26E−9), and transport of small molecules (25 genes, *p* = 3.89E−2) (Figure 3B and Table S3), which were highly abundant in our screening.

**Figure 4:**
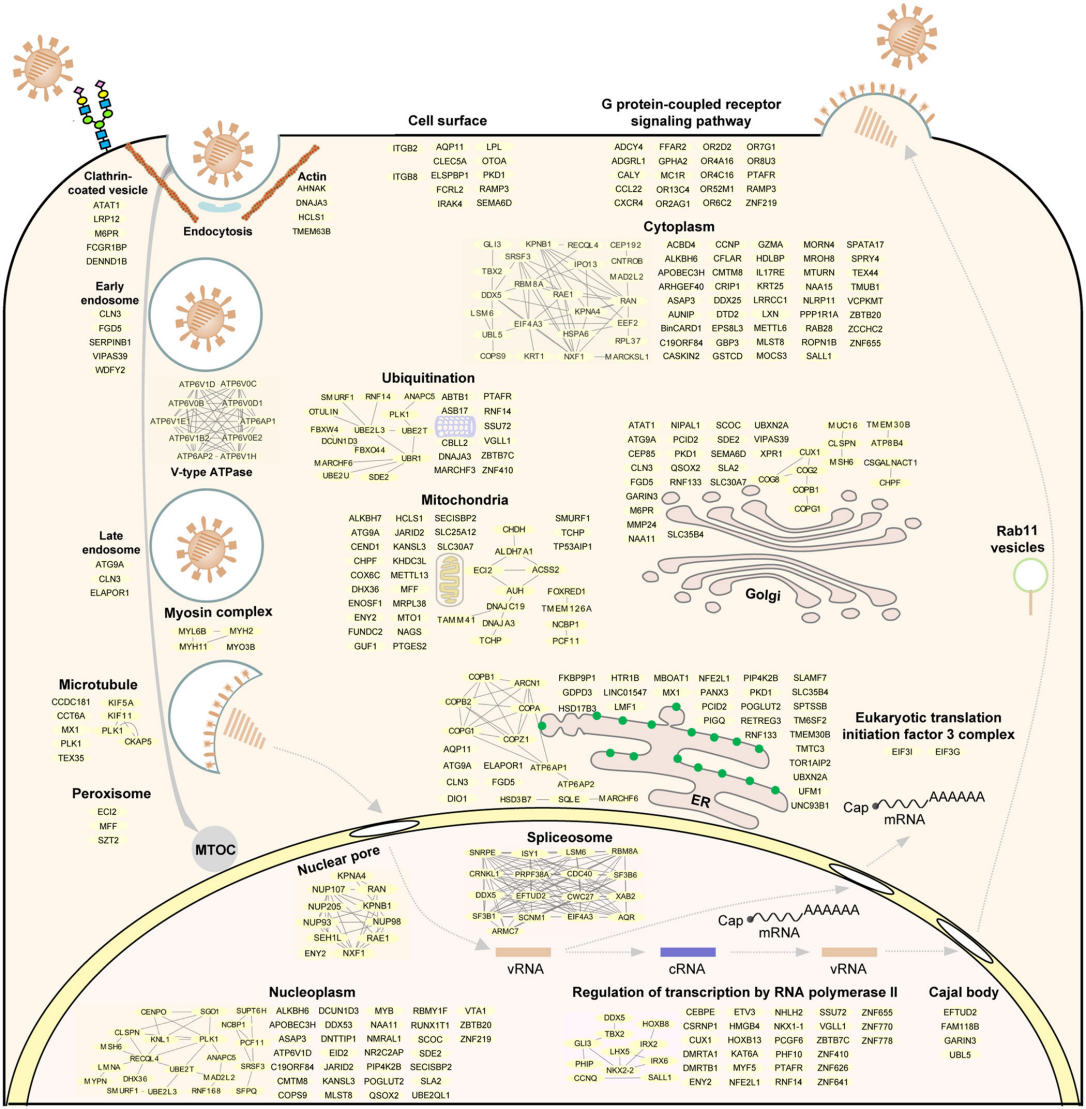
Integrated diagram of the majority of candidate host factors in the life cycle of the H5N1 virus. Candidate genes were analyzed using a database from Gene Ontology (biological process, cellular component, and molecular function) and reactome of the Database for Annotation, Visualization, and Integrated Discovery (DAVID) website, and then mapped to the position most likely to be related to the viral life cycle.

Collectively, these candidate host factors were involved in multiple interactive biological pathways and/or complexes, which might constitute or be associated with the must‐have elements of the viral life cycle (Figure 4 and Table S4). These data provide an important basis for further investigation of the role of the candidate host factors in IAV replication.

### Comparative analysis of five independent genome‐wide siRNA library screenings for human genes involved in the IAV replication cycle

**Figure 5:**
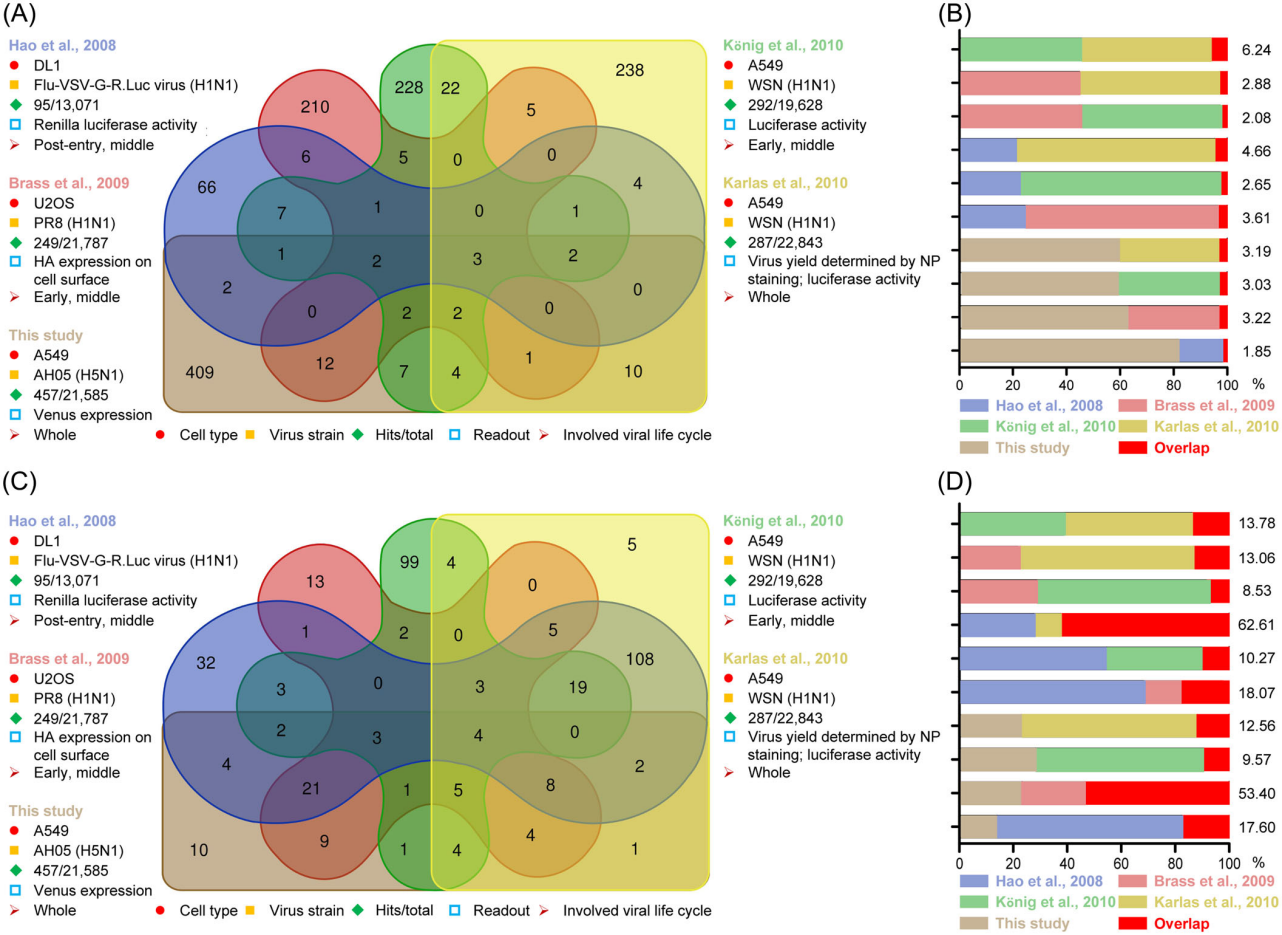
Overlapping rates of candidate genes and their associated pathways in different siRNA library screenings. (A) Intercomparison of candidate genes identified in the five independent genome‐wide siRNA library screenings. (B) Overlapping rates of candidate genes based on pair‐wise comparison of the five independent genome‐wide siRNA library screenings^37^, ^38^, ^39^, ^40^. (C) Intercomparison of reactome pathways involved in the five independent genome‐wide siRNA library screenings. (D) Overlapping rates of reactome pathways based on pair‐wise comparison of the five independent genome‐wide siRNA library screenings^37^, ^38^, ^39^, ^40^.

So far, 1250 human genes have been identified as potential host factors involved in regulating IAV replication in five independent genome‐wide siRNA library screenings (Figure 5 and Table S5), representing about 5.79% of all human protein‐coding genes (using the RefSeq total number of 21,585). The first genome‐wide siRNA library screening for IAV, based on Renilla luciferase activity, was described by Hao et al. in *Drosophila* DL1 cells infected with a genetically engineered reporter virus (Flu‐VSV‐G‐R. Luc), and led to the identification of 110 *Drosophila* genes (95 genes corresponding to the human genome) that regulate the post‐entry and middle stages of the IAV replication cycle^37^ (Figure 5A and Table S5). Then, König et al. infected siRNA‐transfected A549 cells with a reporter virus in which the HA ORF was replaced with Renilla luciferase, and identified 292 cellular factors essential for the early and middle stages of the IAV replication cycle^38^ (Figure 5A and Table S5). These two reporter‐based siRNA library screenings focused on the cellular requirements for certain early stages (endocytosis, fusion, and uncoating) and the middle stages (nuclear import of vRNP, synthesis of viral RNAs, viral protein translation, nuclear export, and traffic of progeny vRNP) of the IAV replication cycle, but were not able to identify host factors involved in the late stages of the viral life cycle (assembly/budding/release of progeny virus). Brass et al. performed a single‐round infection screening on siRNA‐transfected U2OS cells infected with the A/Puerto Rico/8/34 (PR8, H1N1) virus, which identified 249 candidate genes from 21,787 human genes by measuring the surface expression level of HA^39^ (Figure 5A and Table S5). Compared with U2OS cells (a type of osteosarcoma cells), human adenocarcinoma A549 cells are considered a better model for in vitro evaluation of IAV replication. Consequently, Karlas et al. conducted an A549 cell‐based genome‐wide siRNA library screening using a two‐step approach: assessing the NP expression level in siRNA‐transfected A549 cells infected with the A/WSN/33 (WSN, H1N1) virus and measuring the luciferase activity of HEK293T cells containing an inducible IAV‐specific luciferase construct after stimulation with the virus‐containing supernatants from the former cells, which resulted in the identification of 287 primary candidate genes from 22,843 human genes^40^ (Figure 5A and Table S5). It is noteworthy that these four genome‐wide siRNA library screenings were all performed using low pathogenic WSN (H1N1) or PR8 (H1N1) virus. Karlas et al. discovered that only 42.86% of the genes that they identified were common in promoting the replication of both low pathogenic H1N1 and highly pathogenic H5N1 influenza viruses^40^.

Given the severe threat posed by the H5 virus and the absence of published siRNA library screenings to identify host factors involved in the replication of the H5 virus, we performed this multiple‐round infection screening on siRNA‐transfected A549 cells infected with a replication‐competent H5N1 NA‐Venus virus, which allowed us to identify 457 candidate genes from 21,585 human genes based on our analysis of the Venus expression level (Figure 5A and Table S5).

We then examined the commonality of the candidate genes identified in the previous genome‐wide siRNA library screenings and those identified in our screening. To avoid misinterpretation due to differences in the gene symbols among the screenings, we first converted all candidate gene symbols into the official symbol used in the Gene Database on the NCBI website. Although there was significant overlap among the human genes in the different screenings, the overlapping rates were relatively low (0.24%–6.24%) for the candidate genes (Table S6). Only 99 genes from 1250 candidate genes were shared between at least two screenings, and only three candidate genes (*ARCN1*, *ATP6AP1*, and *COPG1*) were simultaneously present in the candidate gene lists of all five genome‐wide screenings (Table S6). The two types of genes with the most commonality among these screenings were those encoding the vacuolar H^+^‐ATPase (V‐ATPase) subunits (ATP6AP1, ATP6AP2, ATP6V0B, ATP6V0C, ATP6V0D1, ATP6V0E2, ATP6V1A, ATP6V1B2, and ATP6V1G1) and the coat protein complex I (COPI) proteins (ARCN1, COPA, COPB1, COPB2, COPG1, and COPZ1), which are mainly involved in endosome acidification and vesicle transport, respectively. In addition, even if Karlas et al. and König et al. used the same source siRNA library, the overlapping rate of their candidate genes was still only 6.24% (the highest among the pair‐wise comparisons) (Figure 5B and Table S6). These relatively low overlapping rates of candidate genes among different screenings might reflect differences in the setups of the individual screening systems.

The candidate genes may form complexes or well‐organized signaling networks that synergistically regulate the replication cycle of IAV. Therefore, we conducted a complete signaling pathway analysis for the five genome‐wide siRNA library screenings using the reactome pathway database, and then performed a homology analysis with the Draw Venn Diagram (Figure 5C). The overlapping rates of the signaling pathways enriched by the candidate genes in our study and the others on pair‐wise comparison were significantly increased (9.57%–53.40%) (Figure 5D and Table S7). Consistent with these findings, strong overlapping rates (from 8.53% to 62.61%) were observed among the five screenings on pair‐wise comparison (Figure 5D). Collectively, these different siRNA library screenings led to much higher overlapping rates at the level of signaling pathways than at the level of individual genes, which suggests that host cellular factors may not function in isolation but rather form complex interactive networks to regulate the IAV replication cycle.

### Validation of the candidate host factors identified in our screening

To date, the role of 41 of the 457 candidate host factors in our screening has been validated and reported, including four host factors—free fatty acid receptor 2 (FFAR2), cation‐dependent mannose‐6‐phosphate receptor (CD‐MPR, also called M6PR), ankyrin repeat and BTB domain containing 1 (ABTB1), and Bcl10‐interacting protein with CARD1 (BinCARD1)—which were thoroughly investigated in our laboratory^41^, ^42^, ^43^, ^44^. Our data demonstrated that FFAR2 positively regulates the endocytosis of IAV via the FFAR2‐β‐arrestin1‐AP2B1 signaling cascade; M6PR interacts with the HA2 subunit of IAV to facilitate the fusion of the viral envelope and the endosomal membrane; ABTB1 promotes the nuclear import of the vRNP complex by counteracting the destabilizing effect of TRIM4 on the viral NP protein; and IAV uses BinCARD1 to facilitate the nuclear import of the vRNP complex, which can be counteracted by BinCARD1‐mediated activation of RIG‐I innate immune signaling as well as TBK1‐p62 axis‐mediated autophagic degradation of BinCARD1. The elucidation of the importance of FFAR2, M6PR, ABTB1, and BinCARD1 in the IAV life cycle provides direct evidence for the reliability and data quality of our genome‐wide siRNA screening.

In‐depth analysis of the remaining 37 candidate host factors (i.e., excluding FFAR2, M6PR, ABTB1, and BinCARD1) revealed that they were required for different stages of IAV replication, including endosomal acidification and trafficking, nuclear import and export, vRNP complex activity, and RNA splicing, or were associated with immunity and the inflammatory response, or other biological processes. Their regulatory roles in the IAV replication cycle have been well elucidated, as detailed below.

#### Endosomal acidification

V‐ATPases, which are membrane‐embedded protein complexes, are the primary proton pumps responsible for the acidification of endocytic vesicles^45^. Our screening identified 12 V‐ATPase subunits (i.e., ATP6AP1, ATP6AP2, ATP6V0B, ATP6V0C, ATP6V0D1, ATP6V0E2, ATP6V1B2, ATP6V1D, ATP6V1E1, ATP6V1G1, ATP6V1H, and TCIRG1) as potential host factors required for IAV replication (Figure S3A). Among them, knockdown of the ATP6V0D1 and ATP6V0C subunits has been reported to inhibit the acidification‐dependent replication of the WSN (H1N1) virus and reduce the NP expression level in the nucleus^37^, ^40^.

#### Endosomal trafficking

The coatomer of COPI vesicles is required not only for endosomal trafficking^46^ but also for bidirectional protein transport between the ER and Golgi^47^. Six of the seven subunits of the coatomer (i.e., ARCN1, COPA, COPB1, COPB2, COPG1, and COPZ1) were identified in our screening (Figure S3B). As the core components of COPI‐coated vesicles, COPB1 regulates the late trafficking of HA to the cell surface^39^, and COPG1 and ARCN1 play important roles in the early invasion stage of IAV^38^, ^48^.

#### Nuclear import and export

During IAV infection, importins [e.g., KPNA1 (Importin α5), KPNA2 (Importin α1), KPNA4 (Importin α3), and KPNA6 (Importin α7)] serve as adaptors linking vRNPs, PB2, or NP protein to KPNB1, which form ternary complexes at the nuclear pore complex (NPC) and are transported to the nucleus (Figure S3C)^49^, ^50^, ^51^. The import and export of viral proteins or RNAs also require interactions with components of the NPC, which is composed of approximately 30 multi‐copies of nucleoporins (Figure S3D)^52^. In terms of the role of the host nuclear export machinery in IAV replication, NXF1 can mediate the export of intronless HA mRNA and spliced M2 or unspliced M1 transcripts during IAV infection^53^, and NS1 hijacks NXF1 and RAE1, and downregulates NUP98 expression to impair cellular mRNA export machinery, thereby antagonizing the host immune response^54^. It is noteworthy that two importins (KPNA4 and KPNB1), four NPCs (NUP93, NUP98, NUP107, and NUP205), and two nuclear export factors (NXF1 and RAE1) were identified in our siRNA library screening.

#### vRNP complex activity

The vRNP complex of IAV catalyzes the transcription and replication of the viral genome^55^, ^56^, ^57^. Three host factors identified in our siRNA library screening––MX1, GBP3, and EIF4A3––have previously been revealed to affect vRNP complex activity and viral replication. MX1 disrupts the PB2–NP interaction in vRNPs, thereby impairing the vRNP complex activity^58^, ^59^; GBP3 overexpression reduces the vRNP complex activity, leading to reduced syntheses of viral RNAs and proteins^60^; and EIF4A3 enhances the vRNP complex activity and synthesis of viral RNAs through interaction with the PB2, PB1, and NP proteins^61^.

#### RNA splicing

Our siRNA library screening identified four host factors (i.e., CLK1, SRSF3, SFPQ, and EIF4A3) that have been reported to regulate the splicing or processing of IAV mRNA. The knockdown of CLK1 in A549 cells increases the ratio of spliced to unspliced M mRNA and reduces the replication of the WSN (H1N1) virus. In contrast, SRSF3 knockdown enhances the ratio of spliced to unspliced mRNA for both the M and NS segments^62^. SFPQ is essential for the production of viral mRNA by increasing the efficiency of viral mRNA polyadenylation^63^. EIF4A3 plays a vital role in splicing the M and NS mRNA and mediating the export of the spliced M2 and NS2 mRNA from the nucleus to the cytoplasm^61^.

#### Immunity and inflammatory response

IAV infection can hijack some immune‐related host factors. Several immune‐related host factors identified in our siRNA library screening have previously been reported to be involved in the immune and inflammatory responses during IAV infection, including CXCL16, CCL22^64^, CXCR4^65^, NMB^66^, PAFR^67^, RTF2^68^, DDX5^69^, SSU72^70^, CFLAR^71^, GZMA^72^, and CLEC5A^73^.

#### Others

The other seven of the 41 validated host factors are SERPINB1^74^, UFM1^75^, CRIP^76^, CSF3R^77^, EEF2^78^, COG8^79^, and COX6C^80^, which affect the replication and pathogenesis of IAV through different mechanisms.

Overall, our in‐depth investigation of the roles of the 41 validated host factors in the replication and pathogenicity of IAV provides direct evidence of the quality and credibility of our genome‐wide siRNA library screening. The candidate genes identified represent a wealth of valuable targets for follow‐up studies to unravel their functions in the IAV replication cycle.

## DISCUSSION

IAV is an important zoonotic pathogen that poses a significant threat to animal and human health. Advances in the field of pathogen biology, especially with the aid of reverse genetics techniques, have allowed us to acquire an excellent understanding of the genomic characteristics and viral protein functions of IAV^81^. In contrast, our knowledge of the host cellular factors and machineries involved in IAV replication is relatively limited. To date, a variety of experimental techniques and methods have been used to study protein–protein interactions, such as yeast two‐hybrid system^82^, bacterial two‐hybrid system^83^, tandem affinity purification technology,^84^ co‐immunoprecipitation^85^, GST pull‐down^86^, fluorescence resonant energy transfer^87^, and bimolecular fluorescence complementation^88^. Although a plethora of host cellular factors that interact with IAV components have been identified^81^, various inherent drawbacks remain to be resolved. For example, these experimental methods are not suitable for screening host proteins that have no direct interaction with viral components, and the directly interactive host factors do not always play bona fide regulatory roles in IAV replication. In contrast, genome‐wide high‐throughput screening technologies, such as siRNA library screening and CRISPR/Cas9 screening^37^, ^38^, ^39^, ^40^, ^89^, ^90^, enable researchers to screen indirectly interacting but functionally important host factors involved in IAV replication.

To date, four genome‐wide siRNA library screenings for host factors involved in IAV replication have been successfully performed^37^, ^38^, ^39^, ^40^. However, the IAV strains used in these screenings were low pathogenic H1N1 viruses. The widespread distribution of the highly pathogenic H5N1 avian influenza virus has had devastating effects on the poultry industry and led to occasional infection and death in humans^10^, ^12^. Moreover, the H5N1 virus has the potential to efficiently transmit among humans and cause a new influenza pandemic^91^, ^92^, ^93^. To gain insights into the host factors involved in the replication of H5 AIVs, we performed a genome‐wide siRNA library screen in A549 cells that were transfected with siRNAs targeting 21,585 human genes and subsequently infected with a replication‐competent H5N1 NA‐Venus virus, which ensured that the screened host factors were involved in the entire replication cycle of the H5N1 virus. We successfully identified 457 human genes potentially involved in the replication of the H5N1 virus based on two criteria: an |SSMD| value ≥1.28 and an IR ≥30%, or an |SSMD| value ≥1.28 and an IR ≤‐20%. Of these candidate host factors, we elucidated the role of FFAR2 in virus internalization, M6PR in viral fusion, and ABTB1 and BinCARD1 in the nuclear import of the vRNP complex^41^, ^42^, ^43^, ^44^. In addition, the roles of 37 other host factors that appeared in our candidate host factor list have been reported in previous studies. These findings, therefore, highlight the reliability and value of our siRNA library screening in identifying host factors engaged in the IAV replication cycle, especially H5N1 viruses.

Including our siRNA library screening, there have now been five genome‐wide siRNA library screenings for host factors involved in the IAV replication cycle. The five screenings have identified 1250 genes with potential roles in the IAV replication cycle. Most of the candidate genes have not yet been examined for their effect on IAV replication and merit further investigation in future studies. It is noteworthy that very low overlapping rates were observed among the candidate genes identified in the five screenings. Meta‐analyses revealed that only three candidate genes were common to all five screenings, 3–5 genes were common among four of the five screenings, 3–11 were common to three of the five screenings, and 10–34 were common in pair‐wise comparisons of the five screenings (Table S6). This low overlapping rate was also a common theme among different siRNA library screenings designed to identify genes for HIV‐1 replication^94^. The low‐level overlapping rates of candidate genes among the five siRNA library screenings suggest that the differences in key experimental setups in these screenings may have affected the results. The first four screenings were all performed with the low pathogenic H1N1 influenza virus, whereas our study was performed using the highly pathogenic H5N1 influenza virus. The overlapping rates of candidate genes between our screening and the individual past four studies ranged from 1.85% to 3.22% (Figure 5B). The impact of the viruses used among screenings on the screening results was also reflected in the study carried out by Karlas et al., which showed that 47 and 49 of 168 validated genes were specific to a low pathogenic and a highly pathogenic influenza virus, respectively^40^. These results indicate that different IAVs may possess distinct features for hijacking specific host cellular factors during their life cycle. This virus‐specific phenomenon of host factors was also often revealed in various publications. For example, we found that the downregulation of host factor FFAR2 has a greater effect on impairing the replication of the H5N1 virus than the H1N1 virus^41^, and that the knockout of gasdermin E prevents the pyroptosis caused by the H7N9 virus, but has no effect on the pyroptosis caused by the H5N1 virus^95^.

The genome‐wide siRNA screenings by Hao et al., Brass et al., and König et al. primarily focused on early and middle replication events, whereas we and Karlas et al. endeavored to examine the whole infection stage (Figure 5A). Moreover, the difference in mRNA expression abundance in the nonpermissive cells (*Drosophila* cells, DL1) used by Hao et al., the permissive cells (Osteosarcoma cells, U2OS) used by Brass et al., and the model cells (lung adenocarcinoma cells, A549) used by König et al., Karlas et al., and us may also be an important contributor to the observed disparities. Furthermore, the sequences of the siRNA targeting specific genes in different libraries may not be identical, which could lead to differences in the knockdown efficiency of specific genes. Karlas et al. and König et al. used the same source of siRNA library for their arrayed siRNA screenings, and their lists of primary candidate genes showed the highest degree of concordance (Figure 5B and Table S6). In addition, the chemical modification of the siRNA duplex in a library can reduce off‐target effects. For example, locked nucleic acid chemistry modification of the siRNA library used in our screening enhances the potency and specificity of the screening compared with unmodified and 2′‐O‐methoxylated (2′‐Ome) chemistry. The criteria used to identify candidate genes also varied considerably among the screens: luciferase expression was used in the first screening by Hao et al., the percentage of HA‐positive cells was used in the second screening by Brass et al., the reduction of viral infection was used in König's screen, three parameters (*Z*‐scores <‐2, cell numbers ≥750, and at least two orders of magnitude of the inhibitory control NP) in the screening were used by Karlas et al., and |SSMD| and IR were used in our screening. Collectively, these differences in experimental setup likely contributed to the low overlapping rate of the candidate genes among the five screenings. Hence, extensive validation studies and a systematic evaluation of the functional roles of these individual host factors in IAV replication are vital for understanding the mechanisms of virus–host interaction and the development of anti‐influenza drugs targeting host factors.

Given the low overlapping rate of genes among the screenings, we performed an in‐depth analysis using the reactome pathway database and found that the candidate genes were enriched for multiple important host cellular events, such as immune system, mRNA splicing and RNA metabolism, ion channel transport, interferon signaling, and COPI‐mediated anterograde transport, which coincide with known stages of the IAV life cycle. By pair‐wise comparison, much higher overlapping rates (8.53%–62.61%) were revealed at the pathway level than at the gene level (Figure 5D). Consequently, it appears that different siRNA library screenings are more convergent in the case of identifying common cellular pathways than individual genes within certain pathways.

In conclusion, our genome‐wide siRNA library screening reveals a comprehensive map of 457 candidate genes and relevant cellular pathways that potentially regulate the replication cycle of the H5N1 virus and, as such, represents a valuable data set for future investigations.

## MATERIALS AND METHODS

### Cells and viruses

A549 cells were cultured in F‐12K medium (Life Technologies) supplemented with 10% fetal bovine serum (FBS, Sigma‐Aldrich), 100 U/ml penicillin, and 100 µg/ml streptomycin (Life Technologies) at 37°C in a 5% CO_2_ humidified incubator.

The H5N1 NA‐Venus reporter virus was generated in our laboratory as described previously^33^. All experiments involving the H5N1 NA‐Venus reporter virus were carried out within the enhanced animal biosafety level 3 (ABSL3+) facility in the Harbin Veterinary Research Institute of the Chinese Academy of Agricultural Sciences, with approvals issued by the Ministry of Agriculture and Rural Affairs of China and the China National Accreditation Service for Conformity Assessment.

### Genome‐wide siRNA library

The Silencer Select Human Genome siRNA Library V4 used in this study was purchased from Life Technologies. The library targets 21,585 human genes, including the Human Druggable Genome (9032 genes), the Human Extended Druggable Genome (1383 genes), and the Human Genome Collection (11,170 genes). siRNAs with locked nucleic acid chemistry modification in this library corresponded to each of the 21,585 genes (>98% of genes listed by NCBI), with three unique and nonoverlapping siRNAs provided per target (a total of 64,755 siRNAs), and were plated in 284 384‐well plates.

### siRNA library screening

A total of 5 µl of siRNA (1 pmol) was incubated with 0.15 µl of Lipofectamine RNAiMAX Transfection Reagent (Invitrogen, Carlsbad, CA, USA) in 15 µl of Opti‐MEM (Gibco)/well of a 384‐well plate at room temperature for 20 min. Next, 3000 A549 cells in 80 µl of F‐12K supplemented with 10% FBS and antibiotics were seeded into each well and cultured at 37°C with 5% CO_2_. At 48 h posttransfection, siRNA‐treated A549 cells were infected with 20 µl of the H5N1 NA‐Venus virus (MOI = 0.1) for 24 h. Cells were then fixed with 4% paraformaldehyde (PFA, Solarbio Science & Technology) for 30 min and stained with nuclear DNA dye Hoechst 33342 for 30 min at room temperature. The Operetta high‐content imaging system (PerkinElmer) was used to capture images, which were subjected to cell infection ratio calculation based on the Venus fluorescence intensity using Columbus 2.9.1 software (PerkinElmer). H5N1 NP siRNA (5′‐AAGGAUCUUAUUUCUUCGGAG‐3′) and scrambled siRNA (Silencer Select Negative Control #1 (4390843)) were included in all screening plates as positive and negative controls, respectively.

### Screening criteria of candidate genes

For the identification of candidate genes, two parameters were used: SSMD and IR of virus replication. First, the SSMD‐based method is suitable for repeated screening, insensitive to outliers, and results in a reasonably low false discovery rate and false non‐discovery rate for selecting inhibiting or activating host factors^96^, ^97^. It can also determine whether a gene has a positive or negative regulatory effect on virus replication. The SSMD‐based criteria used to classify the size of siRNA effects are as follows: |SSMD | < 0.25 (extremely weak); 0.5 > |SSMD | ≥0.25 (very weak); 0.75 > |SSMD | ≥0.5 (weak); 1 > |SSMD | ≥0.75 (fairly weak); 1.28 > |SSMD | ≥1 (fairly moderate); 1.645 > |SSMD | ≥1.28 (moderate); 2 > |SSMD | ≥1.645 (fairly strong); 3 > |SSMD | ≥2 (strong); 5 > |SSMD | ≥3 (very strong); and |SSMD | ≥5 (extremely strong). To identify host factors regulating the replication of the influenza virus, we choose |SSMD | ≥1.28 for at least two individual siRNAs as a criterion of a positive gene. However, SSMD also has an inherent limitation: in the case of perfect repeatability of an experiment repeated three times, even a very small inhibitory effect can still lead to a value of |SSMD | ≥1.28. Such host factors may not be important for IAV replication. Consequently, we also introduced the criterion of the IR of virus replication, where IR = [(infection ratio of scrambled siRNA‐treated well − infection ratio of library siRNA‐treated well)/(infection ratio of scrambled siRNA‐treated well − infection ratio of H5N1 NP siRNA‐treated well)]. Ultimately, on the basis of these two criteria, the candidate genes involved in positive regulation would be those with at least two individual siRNAs with an |SSMD | ≥1.28 and an IR ≥30%, and the candidate genes involved in negative regulation would be those with at least two individual siRNAs with an |SSMD | ≥1.28 and an IR ≤−20%.

### Z‐factor

To provide a quality control for the screening assay, we used the Z‐factor to show the well‐to‐well and plate‐to‐plate reproducibility, where Z‐factor = 1 − [(3 × STDEV (infection ratio of scrambled siRNA‐treated well) + 3 × STDEV (infection ratio of H5N1 NP siRNA‐treated well)]/[(average infection ratio of scrambled siRNA‐treated well − average infection ratio of H5N1 NP siRNA‐treated well)]. The Z‐factor‐based criteria for classifying assay quality are as follows: Z‐factor = 1, an ideal assay; 1 > Z‐factor ≥ 0.5, an excellent assay; 0.5 > Z‐factor > 0, a double assay; Z‐factor = 0, a “yes/no” type assay; and Z‐factor < 0, screening essentially impossible^98^.

### Bioinformatics analysis

Candidate genes in different screenings were uniformly converted into official symbols by using the Gene Database on the NCBI website, and then mapped to the individual keywords using a database from GO (biological process, cellular component, and molecular function) and reactome of the Database for Annotation, Visualization, and Integrated Discovery (DAVID) website (https://david.ncifcrf.gov/)^34^. The classifications with a *p* ≤ 0.05 were retained for further analysis. Bubble diagrams were drawn using https://www.bioinformatics.com.cn, a free online platform for data analysis and visualization. The heatmap was analyzed and visualized using Cluster 3.0/TreeView^99^. The venn diagram was drawn using Draw Venn Diagram (http://bioinformatics.psb.ugent.be/webtools/Venn/). The interaction network analysis was carried out using the STRING database (https://cn.string-db.org/)^100^. The schematic diagrams were drawn using multiple software, including ScienceSlides, Cytoscape, and PowerPoint.

## AUTHOR CONTRIBUTIONS

**Guangwen Wang**: Conceptualization (equal); data curation (lead); formal analysis (lead); funding acquisition (equal); investigation (lead); methodology (lead); validation (lead); writing—original draft (equal); and writing—review and editing (equal). **Li Jiang**: Conceptualization (equal); data curation (supporting); formal analysis (supporting); funding acquisition (equal); investigation (supporting); project administration (equal); supervision (equal); writing—original draft (supporting); and writing—review and editing (equal). **Jinliang Wang**: Formal analysis (supporting); methodology (supporting); resources (supporting). **Qibing Li**: Investigation (supporting); validation (supporting); and visualization (supporting). **Jie Zhang**: Investigation (supporting). **Fandi Kong**: Investigation (supporting). **Ya Yan**: Investigation (supporting). **Yuqin Wang**: Investigation (supporting). **Guohua Deng**: Resources (supporting). **Jianzhong Shi**: Resources (supporting). **Guobin Tian**: Funding acquisition (supporting) and resources (supporting). **Xianying Zeng**: Resources (supporting). **Liling Liu**: Investigation (supporting). **Zhigao Bu**: Conceptualization (equal) and resources (equal). **Hualan Chen**: Conceptualization (lead); formal analysis (equal); funding acquisition (lead); project administration (equal); supervision (equal); writing—original draft (equal); and writing—review and editing (lead). **Chengjun Li**: Conceptualization (lead); data curation (equal); formal analysis (equal); funding acquisition (equal); project administration (lead); supervision (lead); writing—original draft (lead); and writing—review and editing (lead).

## ETHICS STATEMENT

This study did not involve any experiments on animals or humans.

## CONFLICT OF INTERESTS

The authors declare no conflict of interest.

## Supporting information

Supporting information.

Supporting information.

## Data Availability

All the data from this study are available in the main text or supplementary materials.
