# Supplementary material for: Genome‐wide siRNA library screening identifies human host factors that influence the replication of the highly pathogenic H5N1 influenza virus
Source: mLife. 2025 Feb 24;4(1):55–69. doi: 10.1002/mlf2.12168 (PMC11868839; doi:10.1002/mlf2.12168)
Supplement: Supplementary file 2 — Supporting information. [file MLF2-4-55-s002.doc]

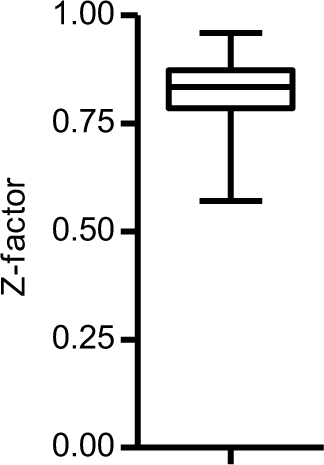


**Figure S1.** Z-factor of all screening plates. Z-factor is used to evaluate the quality of the screening assay, which provides a way to examine the well-to-well and plate-to-plate reproducibility.


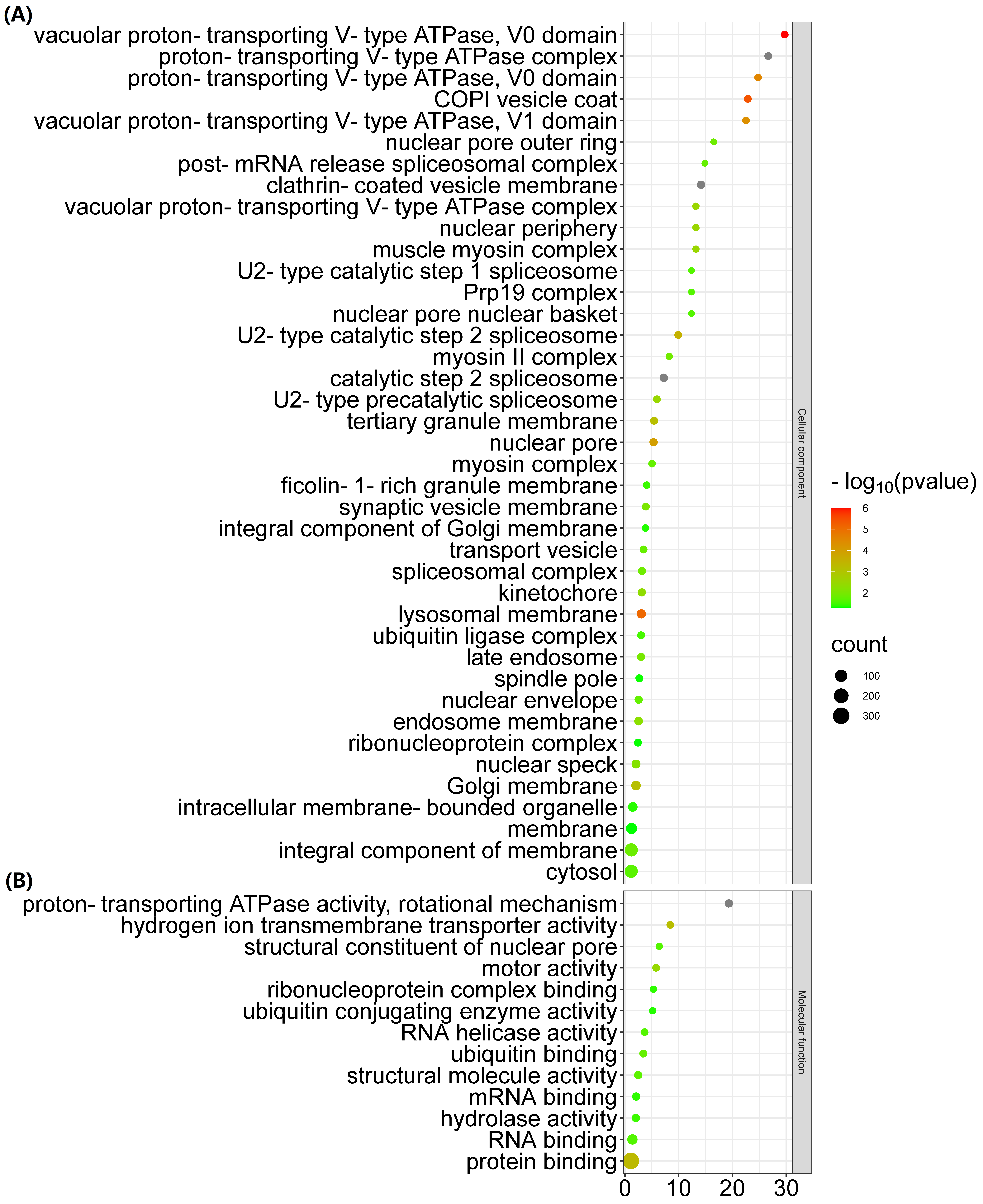


**Figure S2.** The cellular component and molecular function enrichment analysis of candidate host factors. Only p values of the classification ≤ 0.05 are shown. The Y-axis shows the cellular component classification (A) and molecular function classification (B), and the X-axis shows the enrichment score. The colour and size of the bubble represent the range of -log10 (p value) and the number of genes involved in this classification.


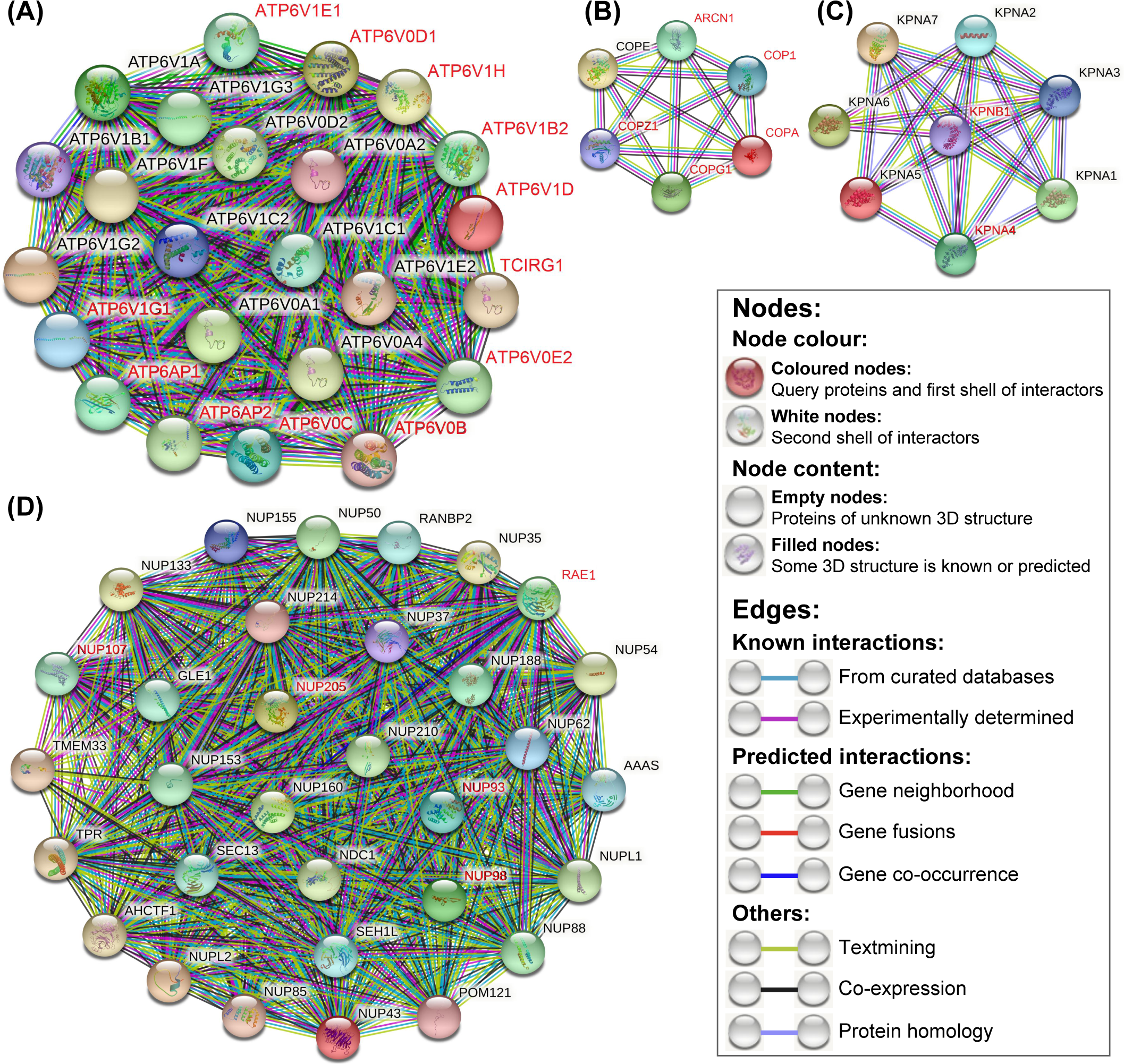


**Figure S3.** Interaction networks of candidate host factors associated with the V-ATPase complex (A), COPI-coated vesicles (B), importins (C), and nuclear pore complex (D), as analyzed by using the STRING database (<https://string-db.org/>). Candidate host factors in red were identified in our genome-wide siRNA library screening.
